# Status, Risk, and Production Practices of Local Sheep and Goat Breeds in Saudi Arabia: Insights from a Breeder Survey

**DOI:** 10.3390/ani16101544

**Published:** 2026-05-18

**Authors:** Abdulrahman S. Alharthi, Ibrahim A. Alhidary, Riyadh S. Aljumaah, Hani H. Al-Baadani, Marimuthu Swaminathan, Ali Al-Shaikhi, Mamdouh Alsharari, Turki M. Alrubie, Markos Tibbo, Abdulkareem M. Matar, Mohammed A. Al-Badwi, Kakoli Ghosh, Nizar Haddad

**Affiliations:** 1 Department of Animal Production, College of Food & Agriculture Science, King Saud University, Riyadh P.O. Box 2460, Saudi Arabia; ialhidary@ksu.edu.sa (I.A.A.); rjumaah@ksu.edu.sa (R.S.A.); hsaeed@ksu.edu.sa (H.H.A.-B.); abdmatar@ksu.edu.sa (A.M.M.); malbadwi@ksu.edu.sa (M.A.A.-B.); 2 Food and Agriculture Organization of the United Nations (FAO), Abdul-Aziz Road, Riyadh 11421, Saudi Arabia; marimuthu.swaminathan@fao.org (M.S.); kakoli.ghosh@fao.org (K.G.); nizar.haddad@fao.org (N.H.); 3 Ministry of Environment Water and Agriculture, Riyadh 11195, Saudi Arabia; ali.alshaikhi@mewa.gov.sa (A.A.-S.); mamalsharari@mewa.gov.sa (M.A.); 4 National Livestock and Fisheries Development Program, Riyadh 12244, Saudi Arabia; talrubie@nfdp.gov.sa; 5 Food and Agriculture Organization of the United Nations (FAO), Abu Dhabi 62072, United Arab Emirates; markos.tibbo@fao.org

**Keywords:** small ruminant, livestock policy, socioeconomic constraints, genetic characterization

## Abstract

Genetic resources of small ruminants are essential for food security in arid regions; however, basic data for each breed in Saudi Arabia remain incomplete. This study establishes a comprehensive national database through a systematic survey of 104 farms, covering 21,214 heads of livestock (sheep and goats) across the kingdom’s primary agro-ecological zones between January and October 2025. Although national census data indicate that major breeds of sheep such as Naeemi, Najdi, Arabi, and Harri or goats such as Ardi exceed the FAO’s numerical thresholds for “not at risk,” our analysis reveals a fundamental paradox of “genetic vulnerability,” defined as a high risk of inbreeding depression and genetic stagnation despite high census numbers. The results show significant regional variations in prolificacy (*p* < 0.05), with the southern region displaying a substantial productivity gap compared to the central and eastern regions, mainly due to reliance on traditional grazing (46.7%) and limited infrastructure. This vulnerability is driven by a high risk of systematic inbreeding, with 65.7% of breeders acquiring sires from their own herds, a situation worsened by a severe 80% shortage of high-quality breeding males in the central region. Furthermore, selection criteria heavily emphasize esthetic phenotypic traits (over 80%) rather than production indicators (less than 8%), hindering genetic progress. Correlation analysis showed that higher farmer education levels were negatively associated with reproductive challenges (r = −0.216), while high feed prices remained a near-universal obstacle (97.1%). To mitigate these risks, we recommend implementing region-specific sire exchange programs to break closed breeding loops and establishing a national performance recording system to shift selection focus from phenotypic traits to measurable productivity. This study provides a vital, evidence-based framework for transitioning toward data-driven, resilient conservation and breeding strategies.

## Simple Summary

Small ruminants (sheep and goats) are essential to Saudi Arabia’s food security and cultural heritage due to their unique adaptation to desert environments. Although official census data show that major breeds like the Najdi and Harri remain numerically stable, this study reveals a hidden genetic vulnerability that threatens their long-term health. By surveying 104 farms and 21,214 animals across the kingdom, we found that widespread traditional practices—specifically, heavy reliance on within-herd breeding and selection based on phenotype rather than actual production traits—are creating significant inbreeding risks and limiting the industry’s economic potential. Additionally, ongoing challenges such as high feed costs continue to strain local farmers. This research provides an evidence-based framework to guide the sector’s transition from traditional management to data-driven breeding strategies, ensuring the preservation of these vital genetic resources for future generations.

## 1. Introduction

The small ruminant sector is a cornerstone of the agricultural economy and food security in Saudi Arabia, providing high-quality protein and supporting rural livelihoods [1,2,3]. Indigenous breeds possess unique adaptations to hyper-arid environments, including superior heat tolerance and water-use efficiency [4]. While Saudi Arabia shares these challenges with other arid regions, its conservation strategies are at a critical juncture [5]. Compared to advanced conservation frameworks in the European Union or Australia, which utilize centralized genomic databases and performance-recording systems, management in Saudi Arabia and similar pastoral regions in the Middle East and North Africa (MENA) have historically relied on numerical census data alone [6]. This lack of integrated phenotypic and genetic data presents a significant barrier to evidence-based policy [7,8].

Although aggregate livestock numbers are often recorded, detailed information on the geographic distribution, population structure, and conservation status of specific native breeds, such as the Najdi, Harri, Naimi, and Arabi sheep, or the Ardi and Saheli goats, remains fragmented or outdated. This data gap presents a significant challenge for policymakers and researchers aiming to develop evidence-based national livestock strategies. Without accurate census and phenotypic data, it is difficult to identify breeds at risk of extinction or to design effective genetic improvement programs that utilize the best-adapted local genotypes [9].

Recent advancements in livestock genomics have increased the urgency of this characterization effort [10]. Genomic analysis of local sheep populations in the Kingdom of Saudi Arabia shows that the Najdi breed has the highest inbreeding levels among regional breeds, with a run of homozygosity-based inbreeding coefficient of 0.053 and a heterozygosity-based inbreeding coefficient of 0.092 [11,12]. These findings indicate that even populations considered numerically stable may face genetic vulnerability due to improper breeding management, such as excessive use of within-herd males [13]. Inbreeding depression can cause reduced fertility, increased disease susceptibility, and a decline in overall productivity, undermining the economic viability of small ruminant farming [14].

Furthermore, significant regional productivity gaps, as shown by twinning rates exceeding 50% in the Central region (Qassim) compared to significantly lower rates in the Southern region (Jazan), are driven by differing environmental and nutritional conditions [15]. While 97.1% of farmers identify high feed costs as a primary barrier, the impact varies by region. The Central plateau benefits from more intensive systems with stabilized total mixed ration protocols. In contrast, productivity in the Southern region is limited by the seasonal volatility of subtropical rangeland forage and high ambient humidity, which increase heat stress and reduce the energy available for reproductive performance. These disparities confirm that regional productivity is not solely determined by genetics, but results from a complex interplay of socioeconomic constraints, localized climate, and the availability of high-quality forage resources [16].

By integrating genetic status with socioeconomic factors, this research offers an evidence-based framework for transitioning to sustainable livestock intensification. The main focus of this study is to evaluate the hypothesis that numerical population stability (FAO ‘not at risk’ status) masks significant underlying genetic vulnerability and productivity constraints in Saudi Arabia’s indigenous small ruminants. These challenges are driven by high within-herd inbreeding risks, a lack of taxonomic characterization for nearly 1.7 million animals, and regional socioeconomic disparities in management and infrastructure [17].

To ensure a standardized assessment of conservation priorities, this study evaluated Saudi small ruminant breeds using the FAO Global Data Bank for Animal Genetic Resources risk categories. These categories range from not at risk to critical, endangered, and extinct, and they are primarily determined by the number of breeding females and the size of the active sire population [18]. Currently, major indigenous breeds, such as Najdi, Harri, and Naimi sheep, as well as Ardi goats, are classified as not at risk because their census populations exceed the FAO threshold of 1000 breeding females [19]. However, this study argues that these classifications may conceal underlying genetic vulnerability. By applying the FAO’s secondary criteria, which consider the rate of inbreeding and the number of herds [20], this research shows that despite high population counts, the lack of structured breeding management places these not-at-risk populations at a higher functional risk than what current statistics indicate.

This study aimed to establish a comprehensive genetic and socioeconomic baseline for the Saudi Arabian small ruminant sector to evaluate the functional status of indigenous resources. The core focus is to investigate hidden genetic vulnerability, where numerical stability may conceal underlying threats to genetic health. To this end, the research prioritized a large-scale assessment of breeding populations for key sheep (Naimi, Najdi, Harri, and Arabi) and goat (Ardi and Gabli) breeds, benchmarking these figures against FAO risk thresholds and characterizing the 1.7 million ‘Undefined’ animals to address gaps in the national inventory. Secondary priorities included mapping socioeconomic constraints—specifically feed costs, inbreeding management, and infrastructure deficits—to identify barriers to modern production. By synthesizing these objectives, the study provides empirical evidence necessary for a tiered policy approach, focusing on structured breeding and targeted extension services to ensure the long-term sustainability of the Kingdom’s animal genetic resources.

## 2. Materials and Methods

### 2.1. Ethical Approval

The research protocol received ethical approval from King Saud University through the Standing Committee on Scientific Research Ethics (ethical number: SE-21-758).

### 2.2. Study Area and Geographic Scope

**Figure 1:**
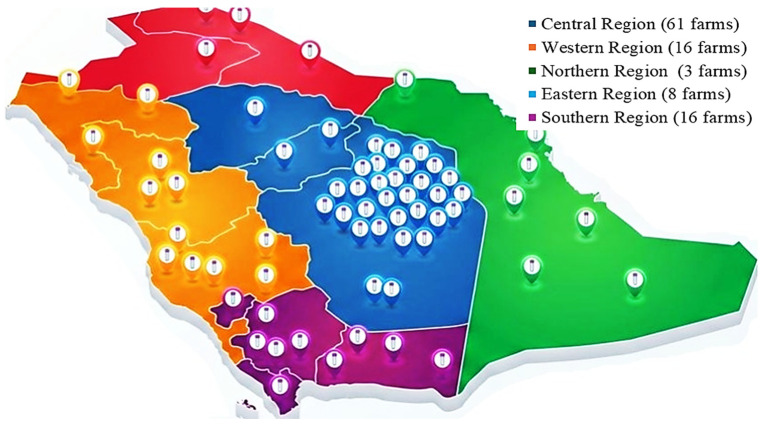
104 farms from different regions in the Kingdom of Saudi Arabia that were surveyed.

The study area was divided into five primary regions based on the administrative boundaries and agro-ecological characteristics established by the Ministry of Environment, Water, and Agriculture (MEWA). This division ensures that the survey captures the environmental diversity of the Kingdom, ranging from the high-altitude central plateaus to the humid southern coastal plains, as shown in Figure 1:

- Central Region: Comprising the agricultural hubs of Riyadh and Al-Qassim (including Al-Kharj, Al-Hariq, Al-Dilam, Al-Dawadmi, Al-Muzahmiya, Dhurma, Shaqra, and Al-Majma’ah). This region is the Kingdom’s primary livestock production center, with a high density of intensive systems.
- Western Region: Including Makkah, Madinah, Jeddah, and Taif, representing the western highlands and coastal transit zones.
- Eastern Region: Including Al-Nu’ayriyah, Al-Khafji, and Dammam, characterized by extensive desert grazing and proximity to major trade ports.
- Northern Region: Covering Tabuk, Arar, and Sakaka, representing the cooler, arid rangelands.
- Southern Region: Encompassing Jazan, Najran, and Al-Baha, characterized by subtropical climates and traditional small-scale production systems.

### 2.3. Sampling Design and Data Collection

A large-scale survey was conducted, covering 104 farms and 21,214 animals (15,319 sheep and 5895 goats) distributed across multiple cities within the main regions of the Kingdom of Saudi Arabia.

To ensure statistical power, the minimum sample size (*n*) was determined using the Cochran [21] formula (n = (*Z*^2^ * *p* (1 − *p*)/*e*^2^), as adapted for livestock populations by Elzarei [22], where *Z* = 1.96, *p* = 0.5, and *e* = 0.05. A target of 384 animals per breed was set to achieve a 95% confidence level. Data collection followed a standardized field protocol: enumerators conducted on-site physical counts and breeder interviews using a structured, pilot-tested questionnaire. Each animal was aged via dental examination to confirm inclusion in the adult breeding population (>1 year).

National breed-specific population estimates were derived using a two-stage extrapolation framework. In Stage 1, breed- and region-specific proportions were calculated based on data from 21,214 animals surveyed from 104 core farms in the primary study areas. In Stage 2, these proportions were applied to the official 2023 Livestock Population Statistics provided by the General Authority for Statistics, focusing specifically on breeding males and females over one year of age. The conservation status of each local breed of sheep (Naeemi, Najdi, Arabi, and Harri) and goats (Ardi) was evaluated using the Food and Agriculture Organization Risk Categories for species with low reproductive rates. A breed was only classified as “secure” (not at risk) if the estimated breeding population exceeded 12,524 breeding females and 629 breeding males. Animals that could not be assigned to a specific recognized breed were categorized as “Undefined” to highlight gaps in the genetic inventory.

### 2.4. Socioeconomic and Production System Analysis

Farmer age, education level, and experience were recorded to evaluate technology readiness. Data on monthly income, feed costs, and commercial orientation (commercial vs. subsistence) were collected to identify financial barriers. Management practices, including production intensity (intensive, semi-intensive, and extensive) and housing types (open, semi-closed, and closed), were categorized to assess climate resilience.

To ensure the reliability of these metrics, all data were subjected to a triangulation validation protocol, in which farmer-reported information was cross-referenced with on-site phenotypic observations and a review of available farm records (e.g., vaccination logs and active males purchase history). In addition, genetic and reproductive metrics were evaluated, such as the percentage of breeding males sourced from within the farmer’s own herd versus external purchases (males sourced from within the herd/total males × 100), to estimate inbreeding risk. Female-to-male ratios (total breeding females/total active males) were calculated by region and breed to assess breeding efficiency. Reproductive efficiency was measured by twinning rates (twins and triplets), defined as the number of offspring born per parturition (total births/number of lambings).

Regional prevalence and distribution of common small ruminant pathologies were quantified by the frequency of clinical symptom outbreaks during a 12-month production cycle. The reliability of these health data was ensured by physical inspection of the flock for clinical signs of respiratory, enteric, or systemic diseases throughout the survey period. Mortality rate was measured as the percentage of lambs or kids lost from birth to weaning within the last 12-month production cycle. Finally, breeders’ selection criteria were evaluated using specific quantitative indicators in accordance with FAO standard guidelines, including: (i) structural stature (height at withers and chest depth); (ii) coat characteristics (hair or wool length and density); (iii) head conformation (ear shape and length). Survey respondents ranked these physical traits against objective performance metrics to determine the percentage of farmers who prioritize appearance over functional productivity.

### 2.5. Statistical Analysis

Data were analyzed using IBM Statistical Package for the Social Sciences version 26.0 (SPSS; Armonk, NY, USA: IBM Corp., 2019). Before analysis, the data were screened for completeness and outliers. Given the nature of the survey data and the presence of ordinal and non-normally distributed variables, both descriptive and non-parametric inferential statistics were used.

Descriptive statistics, including frequencies and percentages, characterized the demographic profile of the farmers, the distribution of small ruminant breeds, and the prevalence of specific production systems. To analyze production constraints, a weighted mean scoring system was applied. Farmer perceptions regarding 13 distinct obstacles were recorded on a 5-point Likert scale (from “Strongly Disagree” to “Strongly Agree”) and ranked by their mean scores to identify the most significant barriers to productivity.

Pearson’s chi-square (*χ*^2^) tests were used to examine the associations between categorical variables.

Because the dependent variables, such as mortality rates (sheep/goats), twinning rates, and overall challenge levels, did not meet the assumptions of normality, non-parametric tests were applied.

Kruskal–Wallis H Test: This test determined whether significant differences existed in performance metrics (mortality and twinning rates) and the “level of challenges” across the five primary geographic regions (Central, Western, Northern, Eastern, and Southern).

To identify the strength and direction of relationships between continuous and ordinal variables (e.g., years of experience, education level, farm size, and mortality rates), Spearman’s Rho rank correlation was calculated. For all statistical tests, a *p* < 0.05 was considered statistically significant. Highly significant results (*p* < 0.01) are specifically highlighted to indicate strong evidence of association or difference.

## 3. Results

### 3.1. Geographic Distribution and Population Density of Indigenous Breeds

The geographic distribution and population density are presented in Table 1. A survey of 104 farms revealed distinct geographic concentrations for various indigenous breeds. The Naimi (mean 110.42) and Harri (mean 113.13) sheep breeds showed a notable presence in the Central Region, while the Najdi breed maintained a significant presence across the Central, Northern, and Western regions. Among goat breeds, the Ardi was widely distributed across the Central, Western, and Eastern regions, whereas the Saheli and Gabli breeds were more localized to the Northern and Southern regions. Statistical analysis indicated that, despite these regional variations, population densities for major breeds did not differ significantly between regions (*p* > 0.05), except for the “Other” goat category.

### 3.2. Regional Variation in Reprodcutive Performance

Regional variations in reproductive performance are presented in Table 2. Reproductive efficiency, measured by multiple birth rates (including twins and triplets), showed highly significant regional disparities (*p* < 0.05 for sheep; *p* < 0.01 for goats). The Eastern Region recorded the highest twinning rate for sheep (mean rank 52.79), whereas the Western Region had the highest goat twinning rate (mean rank 65.71). Conversely, the Southern Region (Jazan) exhibited the lowest twinning rates for both sheep (25.38) and goats (20.14), highlighting a critical productivity gap in that area.

### 3.3. Distribution of Livestock Production System

Distribution of livestock production systems across different administrative regions (Table 3). Production systems varied significantly by region (*p* = 0.001), reflecting different levels of intensification. The Central Region was dominated by intensive (65.4%) and intensive semicircle (85.7%) systems. In contrast, the Southern Region relied heavily on traditional full grazing (46.7%) and semi-intensive (37.5%) methods.

### 3.4. Animal Hosing Types

The characterization of animal housing types by geographic region is presented in Table 4. Housing infrastructure reflected regional management trends, showing a highly significant association (*p* = 0.001). Open housing was the most prevalent type nationally, especially in the Western (23.5%) and Central (54.4%) regions. Hybrid systems were reported only in the Central Region, while semi-closed housing was most common in the Southern Region (47.4%).

### 3.5. Major Reporductive Challenges and Breeding Constraints

The regional prevalence of major reproductive challenges and breeding constraints is presented in Table 5. Inbreeding was identified as a major reproductive challenge in all regions, particularly in the Central (57.8%) and Southern (25.0%) regions. The Central Region also reported a significant shortage of high-quality breeding rams (80%) and low pregnancy rates (51.9%). These findings are consistent with the high rate of within-herd male usage (65.7%) observed in a broader study.

### 3.6. Farmers’ Preferred Phenotype Traits for Breeding Male Selection

The frequency distribution of preferred phenotypic traits for breeding male selection is presented in Table 6. Breeding selection is primarily based on physical appearance rather than production metrics. For both sheep (40.4%) and goats (39.4%), skeletal size was the most preferred trait. For goats, hair length (30.8%) and chest depth (25%) were also important factors, whereas actual production performance and twinning ability were among the least prioritized traits (less than 8%).

### 3.7. Farmers’ Preferred Traits for the Selection of Breeding Stock Across Regions

Regional preferences for selection traits in small ruminant breeding programs are presented in Table 7. Regional analysis confirmed that skeletal size is the dominant selection criterion across the Kingdom, particularly in the Central Region for both sheep (67.24%) and goats (66.67%). There was a significant regional association with trait preference (*p* < 0.05), with the Central Region placing greater emphasis on head characteristics and hair density than other regions.

### 3.8. Relationship Between Farmers’ Socioeconomic Status and Production Challenges

The correlation matrix of socioeconomic factors, breeding experience, and production challenges is presented in Table 8. Correlation analysis showed that years of experience were negatively correlated with the level of education (r = −0.263, *p* < 0.01). Higher levels of education were associated with fewer reproductive challenges (r = −0.216, *p* < 0.05), whereas farmers with more experience often faced more reproductive-related issues (r = 0.195, *p* < 0.05). Larger sheep flock sizes were positively correlated with years of experience (r = 0.214, *p* < 0.05).

### 3.9. Common Disease Challenges in the Surveyed Farms

The regional prevalence and distribution of common small ruminant pathologies are presented in Table 9. Respiratory diseases were the most frequently reported health issue (*p* = 0.02), especially in the Central (48.44%) and Southern (18.75%) regions. Other significant pathologies included enterotoxemia and septicemia, both with the highest prevalence in the Central Region. Reproductive and metabolic disorders also represented a significant burden, particularly for farmers in the Central Region.

### 3.10. Major Challenges Faced by the Farmers in Managing Small Ruminants

The relative distribution of opinions among those surveyed regarding the most important obstacles facing sheep and goat farming is presented in Table 10. The most significant barrier to sheep and goat farming was the rise in feed prices, with 97.11% of respondents agreeing or strongly agreeing that it is a major obstacle. Other critical constraints included the high cost of building modern shelters (78.85%), rising labor costs (70.19%), and inadequate guidance or extension services (71.16%).

### 3.11. Perception Levels of Challenges Across Studied Regions

The assessment of perceived challenge levels across different geographic regions is presented in Table 11. The perception of challenge levels differed significantly across regions (*p* = 0.001 in the Central Region). Farmers in the Eastern (87.5%) and Western (68.8%) regions reported the highest levels of agreement regarding the severity of challenges they face. In the Central Region, the majority of farmers (60.7%) also agreed that challenges were significant.

### 3.12. FAO Threshhold for Specific Breeds to Categorize Population Risk Status

Breed risk categories based on the population size of breeding males and females are presented in Table 12. Risk classifications were assigned according to standardized FAO criteria for species with low reproductive rates [19]. These criteria use a dual-axis assessment of population trends and the absolute number of active breeding individuals to categorize breeds along a spectrum from ‘Not at Risk’ to ‘Critical,’ as illustrated in the reference framework.

### 3.13. Estimated Breeding Population of Sheep in Surveyed Regions

The estimated national breeding populations of sheep, categorized by breed and region, are presented in Table 13. These data show the distribution of active breeding males and females across the Riyadh, Qassim, and Jazan regions. Estimates were derived using a two-stage extrapolation framework that integrates site-specific survey data with the 2023 national livestock statistics from the General Authority for Statistics.

### 3.14. Estimated Breeding Population of Goats by Breed and Region

The estimated national breeding populations of goats, categorized by breed and region, are shown in Table 14. As with the sheep population analysis, these data represent the estimated census of active breeding males and females in the Riyadh, Qassim, and Jazan regions. The figures were calculated using a two-stage extrapolation framework that combined primary farm survey metrics with the 2023 national livestock statistics. As shown in the table, the disproportionate concentration of certain breeds, such as the Saheli in Jazan and the Ardi in Riyadh, reflects significant regional specialization and highlights the geographic heterogeneity of the Kingdom’s goat genetic resources.

All surveyed sheep and goat breeds exceeded FAO thresholds, indicating no immediate risk. Najdi sheep dominated the central regions (2.41 million females), while undefined goat populations (0.84 million females) were notably high in Jazan and Riyadh. However, the numbers of undefined sheep and goat populations have not yet been characterized.

## 4. Discussion

The small ruminant sector is a fundamental component of regional food security and agricultural sustainability in Saudi Arabia [23,24]. Historically, the selection of indigenous breeds like the Naimi, Najdi, and Harri prioritized environmental resilience over intensive productivity [25]. The finding of this study that major breeds exceed the FAO “not at risk” numerical thresholds is encouraging and aligns with recent reports from the Ministry of Environment, Water, and Agriculture [26]. However, large, undefined populations highlight the urgent need for genetic classification. Furthermore, the data reveal a paradox of “genetic vulnerability” despite high population numbers. This vulnerability is characterized by systemic inbreeding risks, a large “Undefined” population of 1.7 million animals, and significant regional disparities in productivity. Previous studies have often focused on aggregate livestock numbers, leaving a critical gap in breed-specific data. While earlier work by Musthafa et al. [27] highlighted high genetic polymorphism in local sheep, recent genome-wide analyses have warned of “historical isolation” in specific breeds such as the Najdi, which shows significantly higher inbreeding coefficients compared to the Naimi. The transition from the traditional nomadic profiles described in the literature [28] to a more highly educated farmer base represents a significant demographic shift. However, this human capital is currently neutralized by systemic structural barriers. The move toward a data-driven sector is at a critical juncture; widespread reliance on internal sire sourcing, compounded by the extreme economic pressure of feed costs, creates a cycle of genetic isolation. This mirrors the historical isolation detected in recent genomic studies of the Najdi breed [29], suggesting that without a policy shift toward structured sire exchange and performance recording, the sector’s high educational readiness will remain decoupled from actual genetic and economic progress.

The geographic distribution of breeds highlights a distinct regional specialization within Saudi livestock, a characteristic shared with various Middle Eastern pastoral systems [30]. The concentration of Naimi and Harri sheep in the Central Region and the presence of Saheli and Gabli goats in the Northern and Southern regions reflect long-standing cultural preferences and environmental adaptations, as reported in a previous study [31]. For example, the Harri breed adaptation to the volcanic north western terrain enables survival under high thermal stress and poor nutrition [19]. While population densities remain statistically uniform across most regions, this high degree of breed localization suggests that localized environmental or epidemiological shocks could disproportionately affect specific genetic lineages, necessitating targeted regional conservation strategies.

Furthermore, the divergent prolificacy observed between regions highlights a significant productivity gap, likely driven by the interplay of management systems and infrastructure. The superior reproductive efficiency in the Eastern and Western regions contrasts sharply with that of the Southern region (Jazan). This disparity is likely exacerbated by the Southern region’s reliance on traditional extensive grazing and limited access to modern veterinary infrastructure [32]. The shift toward intensive and semi-intensive systems in the Central Region indicates an institutional move toward agribusiness models, moving beyond traditional nomadic paradigms [33]. In contrast, the lower reproductive metrics in the South are inherently linked to extensive grazing systems and the resulting nutritional volatility. Additionally, the nationwide prevalence of open housing reveals a systemic lack of climate-controlled facilities, which are essential for mitigating heat stress in arid environments. The significant association between geography and housing type further suggests that infrastructure modernization remains unevenly distributed across the Kingdom.

The most concerning finding was the systemic risk of inbreeding. With 65.7% of farmers sourcing breeding males from within their own herds, the risk of inbreeding depression—characterized by reduced fertility and increased disease susceptibility—is high. Recent molecular studies by Samara et al. [25] confirmed that although local breeds have high diversity, certain populations, specifically the Najdi, exhibit the longest runs of homozygosity, indicating a high degree of genetic isolation. The Central Region reported a critical shortage of high-quality breeding rams (80%), forcing reliance on suboptimal internal animals. This practice directly undermines the long-term genetic health of even numerically stable populations, such as Najdi sheep. The reproductive potential of Saudi indigenous breeds, particularly the multiple birth rates exceeding 50% in intensive systems in Qassim, demonstrates a genetic capacity that is competitive with advanced global benchmarks [15]. However, a significant gap exists when compared to world-famous breeds like the Boer goat or Dorper sheep, where selection is driven strictly by data-verified growth and prolificacy.

There is a clear misalignment between farmers’ selection criteria and economic productivity. Farmers prioritize skeletal size, height, and hair length over actual production metrics such as twinning ability or growth rates, which were prioritized by fewer than 8% of respondents. This focus on appearance is a known obstacle to livestock improvement in the Middle East; Abouheif et al. [34] noted that consumer preference for the “large-framed” Najdi sheep often drives breeding goals regardless of feed efficiency. This preference for physical stature over measurable performance indicators hinders the development of high-output breeding lines [35]. Educational interventions are needed to shift the focus towards record-based selection to improve flock efficiency.

The correlation analysis provides some optimism: higher levels of education are negatively correlated with reproductive challenges, indicating that educated farmers are more likely to adopt improved management practices. However, more experienced farmers often face greater challenges, possibly due to resistance to changing traditional, less efficient methods. Respiratory diseases are the main health burden, particularly in the Central and Southern regions. Previous epidemiological studies in Saudi Arabia have identified Pasteurella hemolytica as a leading cause of pneumonia in local small ruminants [36]. The high prevalence of enterotoxaemia and septicemia in the Central Region indicates that, even in more intensive systems, biosecurity and vaccination protocols remain inadequate.

The overwhelming consensus (97.11%) that rising feed prices are the primary obstacle confirms that the sector’s current economic model is fragile. These difficulties are deeply rooted in local structural constraints, specifically the reliance on ‘Undefined’ local genetic sources (1.7 million animals) that lack formal characterization and the systemic shortage of high-quality breeding stock (80% in the Central region). This aligns with the findings of Wanyoike et al. [37] in similar arid regions, where feed costs are the largest variable expense and a major driver of herd liquidation during droughts. This challenge is further compounded by local difficulties such as high labor and construction costs and a lack of climate-controlled infrastructure to mitigate extreme heat stress. The perception of these difficulties is highest in the Eastern (87.5%) and Western (68.8%) regions, indicating that, even in high-productivity areas, financial margins are extremely narrow.

Despite the comprehensive scope of this Saudi Arabia survey, several limitations must be acknowledged. First, although the triangulation validation protocol ensured the reliability of production cycle data during study periods, certain longitudinal metrics, such as long-term mortality and reproductive trends, partly relied on breeder recall, which may introduce recall bias. Second, the cross-sectional design provides a vital snapshot of the sector’s current status but does not account for inter-annual climatic variability or changing market dynamics over multiple years. Third, while our findings quantify genetic vulnerability using management and demographic proxies, the study did not employ molecular genotyping to confirm the precise level of heterozygosity within the 1.7 million animals classified as undefined. Finally, although the sample size of 104 farms is robust for a specialized regional study, expanding future research to include more remote pastoral units could further refine our understanding of the Kingdom’s most isolated genetic lines. Despite these constraints, this research provides the first integrated socioeconomic and phenotypic baseline required for the strategic modernization of Saudi Arabia’s small ruminant sector.

## 5. Conclusions

This study critically assesses the small ruminant sector in Saudi Arabia and notes that although local sheep and goat breeds demonstrate numerical stability according to FAO standards, they face significant genetic vulnerability. The widespread use of breeding males within flocks (65.7%) presents a structural risk of reduced fitness due to inbreeding, which could ultimately result in a decline in essential breed traits, such as heat tolerance and water use efficiency, both crucial for climate change adaptation. The research also highlights the productivity gap between intensive production systems in the central region and traditional grazing systems in the south. With 91% of farmers struggling with high feed costs and over 70% lacking adequate advisory services, the sector’s sustainability is under considerable strain. To ensure the long-term sustainability of these animal genetic resources, the Kingdom must prioritize the scientific classification of 1.7 million unclassified animals and establish organized national breeding programs. By combining the high human capital of a skilled farming base, targeted digital expansion, and improved infrastructure, Saudi Arabia can transform this traditional sector into a modern, flexible, and highly productive component of its national food security strategy.

## Figures and Tables

**Table 1:** Geographic distribution and population density of indigenous sheep and goat breeds in Saudi Arabia.

Breed	The Area	Mean Rank	Mean	Kruskal–Wallis Test	*p*-Value
		Sheep breeds			
Naimi	Central Region	7.44	110.42	3.291	0.193
	Northern Region	5.00			
	Southern Region	1.00			
Najdi	Central Region	19.19	137.76	2.143	0.343
	Northern Region	15.00			
	Western Region	35.00			
Harri	Central Region	9.65	113.13	4.460	0.108
	Southern Region	16.36			
	Western Region	12.00			
Araby	Central Region	1.00	321.36	3.392	0.183
	Southern Region	5.00			
	Eastern Region	7.14			
Other	Central Region	6.60	243.27	0.306	0.580
	Southern Region	5.50			
		Goats breeds			
Ardi	Central Region	29.08	64.39	3.718	0.294
	Western Region	29.42			
	Northern Region	8.50			
	Eastern Region	34.42			
Shami	Central Region	4.50	40.50	_	_
Saheli	Northern Region	1.00	31.25	1.8	1.8
	Southern Region	3.00			
Gabli	Central Region	2.00	86.67	0.0	1.0
	Southern Region	2.00			
Other	Central Region	12.12	27.00	7.797	0.099
	Western Region	26.00			
	Northern Region	3.00			
	Eastern Region	5.00			
	Southern Region	16.95			

**Table 2:** Regional variation in reproductive performance (twinning rates) for sheep and goats.

Twin Birth Rate	The Area	Mean Rank	Mean	Kruskal–Wallis Test	*p*-Value
Twin birth rate in sheep	Central Region	40.34	30.1645	10.391	0.034
	Western Region	49.38			
	Northern Region	28.50			
	Eastern Region	52.79			
	Southern Region	25.38			
Twin birth rate in goats	Central Region	44.77	49.5349	30.568	0.001
	Western Region	65.71			
	Northern Region	8.00			
	Eastern Region	52.29			
	Southern Region	20.14			

**Table 3:** Distribution of livestock production systems across different administrative regions.

Production System								
Region	Production System	Intensive	Intensive Semicircle	Semi-Intensive	Full Grazing	Total	χ^2^	*p*-Value
Central Region	No.	34.0	18.0	4.0	5.0	61.0	51.89	0.001
	%	65.4	85.7	25.0	33.3	58.7		
Western Region	No.	14.0	0.0	1.0	1.0	16.0		
	%	26.9	0.0	6.3	6.7	15.4		
Northern Region	No.	0.0	0.0	2.0	1.0	3.0		
	%	0.0	0.0	12.5	6.7	2.9		
Eastern Region	No.	4.0	0.0	3.0	1.0	8.0		
	%	7.7	0.0	18.8	6.7	7.7		
Southern Region	No.	0.0	3.0	6.0	7.0	16.0		
	%	0.0	14.3	37.5	46.7	15.4		

**Table 4:** Characterization of animal housing types by geographic region.

Region	Types of Housing						*χ* ^2^	*p*-Value
		Open	Semi-Closed	Hybrid System	Closed	Total		
Central Region	No.	37.0	10.0	9.0	5.0	61.0	36.4	0.001
	%	54.4	52.6	100.0	62.5	58.7		
Western Region	No.	16.0	0.0	0.0	0.0	16.0		
	%	23.5	0.0	0.0	0.0	15.4		
Northern Region	No.	2.0	0.0	0.0	1.0	3.0		
	%	2.9	0.0	0.0	12.5	2.9		
Eastern Region	No.	8.0	0.0	0.0	0.0	8.0		
	%	11.8	0.0	0.0	0.0	7.7		
Southern Region	No.	5.0	9.0	0.0	2.0	16.0		
	%	7.4	47.4	0.0	25.0	15.4		

**Table 5:** Regional prevalence of major reproductive challenges and breeding constraints.

Region	Reproduction Challenges						*χ* ^2^	*p*-Value
		Inbreeding	Shortage of High-Quality Breeding Rams	Low Pregnancy Rate	Other	Total		
Central Region	No.	37.0	8.0	14.0	2.0	61.0	27.41	0.007
	%	57.8	80.0	51.9	66.7	58.7		
Western Region	No.	5.0	2.0	9.0	0.0	16.0		
	%	7.8	20.0	33.3	0.0	15.4		
Northern Region	No.	3.0	0.0	0.0	0.0	3.0		
	%	4.7	0.0	0.0	0.0	2.9		
Eastern Region	No.	3.0	0.0	4.0	1.0	8.0		
	%	4.7	0.0	14.8	33.3	7.7		
Southern Region	No.	16.0	0.0	0.0	0.0	16.0		
	%	25.0	0.0	0.0	0.0	15.4		

**Table 6:** Frequency distribution of preferred phenotypic traits for breeding male selection.

The Preferred	No.	%	No.	%
	Ram Sheep		Ram Goats	
Skeletal size	42.0	40.40	41.0	39.40
Height	18.0	17.30	18.0	17.30
Head	17.0	16.30	8.0	7.70
Hair length	11.0	10.60	32.0	30.80
Source/Origin	10.0	9.60	12.0	11.50
Production	6.0	5.80	8.0	7.70
Color	6.0	5.80	12.0	11.50
Neck	5.0	4.80	3.0	2.90
Beauty	4.0	3.80	0.0	0.00
Twins	4.0	3.80	6.0	5.80
Large head	3.0	2.90	3.0	2.90
Hair density	3.0	2.90	3.0	2.90
Chest depth	0.0	0.00	26.0	25.00
Ears	0.0	0.00	5.0	4.80
Other qualities	8.0	7.70	17.0	16.30

**Table 7:** Regional preferences for selection traits in small ruminant breeding programs.

Rigon	The Relative Distribution of Preferred Traits in Selecting Sheep												*χ* ^2^	*p*-Value
	Skeletal Size		Hair Density		Source		Height		Head		Other			
	No.	%	No.	%	No.	%	No.	%	No.	%	No.	%		
Central Region	39.0	67.24	17.0	58.62	9.0	45.00	17.0	58.62	15.0	93.75	26.0	89.66	69.78	0.002
Western Region	3.0	5.17	3.0	10.34	3.0	15.00	3.0	10.34	0.0	0.00	0.0	0.00		
Northern Region	0.0	0.00	0.0	0.00	0.0	0.00	0.0	0.00	0.0	0.00	0.0	0.00		
Eastern Region	8.0	13.79	7.0	24.14	7.0	35.00	7.0	24.14	0.0	0.00	0.0	0.00		
Southern Region	8.0	13.79	2.0	6.90	1.0	5.00	2.0	6.90	1.0	6.25	3.0	10.34		
**The relative distribution of preferred traits in selecting goats**														
Central Region	42.0	66.67	20.0	58.82	14.0	43.75	18.0	64.29	15.0	71.43	20.0	80.00	71.11	0.012
Western Region	9.0	14.29	8.0	23.53	8.0	25.00	5.0	17.86	4.0	19.05	1.0	4.00		
Northern Region	3.0	4.76	0.0	0.00	3.0	9.38	0.0	0.00	0.0	0.00	0.0	0.00		
Eastern Region	6.0	9.52	6.0	17.65	6.0	18.75	4.0	14.29	1.0	4.76	1.0	4.00		
Southern Region	3.0	4.76	0.0	0.00	1.0	3.13	1.0	3.57	1.0	4.76	3.0	12.00		

**Table 8:** Correlation matrix of socioeconomic factors, breeding experience, and production challenges.

		Educational Level	Years of Experience in Breeding	Level of Challenges	Reproduction Challenges	Number of Sheep Owned	Number of Goats Owned
Educational level	rxy						
	*p*-value						
Years of experience in livestock breeding (year)	rxy	−0.263 **					
	*p*-value	0.007					
Level of challenges	rxy	0.144	−0.121				
	*p*-value	0.144	0.223				
Reproduction challenges	rxy	−0.216 *	0.195 *	0.256 **			
	*p*-value	0.028	0.047	0.009			
The number of sheep	rxy	0.033	0.214 *	−0.113	−0.187		
	*p*-value	0.737	0.029	0.254	0.058		
The number of Goat	rxy	−0.153	0.123	−0.040	0.015	0.165	
	*p*-value	0.121	0.214	0.685	0.879	0.094	

**. Correlation is significant at the 0.01 level. *. Correlation is significant at the 0.05 level.

**Table 9:** Regional prevalence and distribution of common small ruminant pathologies.

Region	Respiratory		Enterotoxemia		Septicemia		Mineral Deficiency		Reproductive & Metabolic		Other		*χ* ^2^	*p*-Value
	No.	%	No.	%	No.	%	No.	%	No.	%	No.	%	80	0.02
Central Region	31.0	48.44	28.0	49.12	26.0	50.98	27.0	54.00	22.0	56.41	36.0	52.94		
Western Region	11.0	17.19	10.0	17.54	9.0	17.65	7.0	14.00	8.0	20.51	12.0	17.65		
Northern Region	4.0	6.25	3.0	5.26	2.0	3.92	2.0	4.00	0.0	0.00	1.0	1.47		
Eastern Region	6.0	9.38	5.0	8.77	4.0	7.84	4.0	8.00	2.0	5.13	3.0	4.41		
Southern Region	12.0	18.75	11.0	19.30	10.0	19.61	10.0	20.00	7.0	17.95	16.0	23.53		

**Table 10:** The relative distribution of opinions of those surveyed regarding the most important obstacles facing sheep and goat farming.

The Obstacles Facing Sheep and Goat Farming:	Strongly Disagree	Disagree	Neutral	Agree	Strongly Agree
The rise in feed prices and their limited availability	0.00	1.0	1.92	13.46	83.65
The rising costs of building modern shelters	1.92	5.77	13.46	16.35	62.50
Rising labor costs	1.0	5.77	23.08	17.31	52.88
The difficulty of obtaining medications and veterinary services in the same area	1.92	9.62	14.42	22.12	51.92
The spread of some diseases and the increase in mortality rate	4.81	4.81	25.00	28.8	36.5
Decline in fertility	6.7	26.0	19.2	25.00	23.08
Inadequacy of guidance services	4.81	8.65	15.38	16.35	54.81
Not using the records	9.62	7.69	27.88	22.1	32.7
The difficulty of using artificial insemination in reproduction	6.7	1.9	25.0	17.31	49.04
The difficulty of choosing the right breed	11.54	18.27	29.81	21.15	19.23
The increase in the number of transfers throughout the year	7.69	5.77	33.65	23.1	29.8
Weak growth and low production	13.5	20.2	23.1	29.81	13.46
The lack of good breeding males and animals	13.46	25.96	19.23	21.15	20.19

**Table 11:** Assessment of perceived challenge levels across different geographic regions.

The Area	Level of Challenges	No.	%	SEM	Mean	*χ* ^2^	*p*-Value
Central Region	Disagree	2	3.3	0.090	3.9016	45.033	0.001
	Neutral	12	19.7				
	Agree	37	60.7 **				
	Strongly agree	10	16.4				
Western Region	Agree	11	68.8	0.120	4.3125	2.25	0.134
	Strongly agree	5	31.3				
Northern Region	Neutral	1	33.3	0.333	3.6667	0.333	0.564
	Agree	2	66.7				
Eastern Region	Neutral	1	12.5	0.125	3.8750	4.5	0.034
	Agree	7	87.5 *				
Southern Region	Disagree	1	6.3	0.232	3.9375	3.5	0.321
	Neutral	4	25.0				
	Agree	6	37.5				
	Strongly agree	5	31.3				

* Statistically significant at the 5% probability level (*p* < 0.05). ** Highly statistically significant at the 1% probability level (*p* < 0.01). SEM = Standard Error of the Mean.

**Table 12:** Breed risk categories based on population size of breeding males and females.

Trend	Males (n)	Population Size—Breeding Females (n)						
		≤240	241–360	361–2400	2401–3600	3601–4800	4801–7200	>7200
Increasing trend & >80% pure breeding	≤5							
	6–20							
	21–35							
	>35							
Stable/Decreasing trend or ≤80% pure breeding	≤5							
	6–20							
	21–35							
	>35							

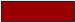
 *Critical* 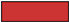 *Endangered* 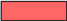 *Vulnerable*  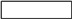 *Not at risk*. Note: This categorization applies to species with low reproductive capacity—horses, donkeys, cattle, yaks, buffaloes, deer, sheep, goats, and camelids.

**Table 13:** Estimated national breeding population of sheep, categorized by breed and region.

Region	Naimi (F/M)	Najdi (F/M)	Harri (F/M)	Arabi (F/M)	Undefined (F/M)
Riyadh	416,447/37,369	1,60,900/123,404	561,825/34,762	62,846/11,298	530,024/9559
Jazan	30,211/4939	0/0	262,836/55,317	78,549/3951	205,435/17,780
Qassim	0/0	803,645/51,539	770,303/51,539	459,882/38,655	137,965/12,885

Note: F = Breeding females, M = Breeding males.

**Table 14:** Estimated national breeding population of goats, categorized by breed and region.

Region	Ardi (F/M)	Shami (F/M)	Saheli (F/M)	Gabli (F/M)	Undefined (F/M)
Riyadh	368,700/21,667	53,546/8125	0/0	38,247/1083	280,732/16,792
Jazan	0/0	0/0	73,368/10,479	0/0	410,863/71,259
Qassim	57,476/20,987	79,278/8745	0/0	0/0	148,646/20,987

Note: F = Breeding females, M = Breeding males.

## Data Availability

All data presented in this trial are available from the corresponding author.
